# *Kdr* genotyping and the first report of V410L and V1016I *kdr* mutations in voltage-gated sodium channel gene in *Aedes aegypti* (Diptera: Culicidae) from Iran

**DOI:** 10.1186/s13071-024-06123-w

**Published:** 2024-01-25

**Authors:** Ahmadali Enayati, Reza Valadan, Mahboobeh Bagherzadeh, Mohammad Cheraghpour, Seyed Hassan Nikookar, Mahmoud Fazeli-Dinan, Nasibeh Hosseini-Vasoukolaei, Farzaneh Sahraei Rostami, Razieh Shabani Kordshouli, Ahmad Raeisi, Fatemeh Nikpour, Abdolreza Mirolyaei, Fatemeh Bagheri, Mohammad Mehdi Sedaghat, Morteza Zaim, David Weetman, Janet Hemigway

**Affiliations:** 1 Department of Medical Entomology and Vector Control, School of Public Health and Health Sciences Research Center, Mazandaran University of Medical Sciences, Sari, Iran; 2 Department of Immunology and Molecular and Cellular Biology Research Center, Mazandaran University of Medical Sciences, Sari, Iran; 3 Department of Medical Entomology and Vector Control, School of Public Health, Student Research Center, Mazandaran University of Medical Sciences, Sari, Iran; 4 Health Sciences Research Center, Department of Medical Entomology and Vector Control, School of Public Health, Mazandaran University of Medical Sciences, Sari, Iran; 5 Department of Medical Entomology and Vector Control, School of Public Health, Mazandaran University of Medical Sciences, Sari, Iran; 6 Vector Borne Diseases Control Department, Iran CDC, Ministry of Health and Medical Education, Tehran, Iran; 7 Department of Medical Parasitology & Mycology, Tehran University of Medical Sciences, Tehran, Iran; 8 Department of Environmental Chemical Pollutants and Pesticides, Institute for Environmental Research, Tehran University of Medical Sciences, Tehran, Iran; 9 Hormozgan Provincial Health Center, Department of Communicable Diseases Control, Hormozgan University of Medical Sciences, Bandar Abbas, Iran; 10 Department of Medical Entomology and Vector Control, Tehran University of Medical Sciences, Tehran, Iran; 11 Vector Biology Department, Liverpool School of Tropical Medicine, Liverpool, UK

**Keywords:** *Aedes aegypti*, Iran, Insecticide resistance, *Kdr*, *Vgsc*

## Abstract

**Background:**

*Aedes aegypti* is the main vector of arboviral diseases worldwide. The species invaded and became established in southern Iran in 2020. Insecticide-based interventions are primarily used for its control. With insecticide resistance widespread, knowledge of resistance mechanisms is vital for informed deployment of insecticidal interventions, but information from Iranian *Ae. aegypti* is lacking.

**Methods:**

Fifty-six *Ae. aegypti* specimens were collected from the port city of Bandar Lengeh in Hormozgan Province in the South of Iran in 2020 and screened for *kdr* mutations. The most common *kdr* mutations in Latin America and Asia (V410L, S989P, V1016G/I and F1534C), especially when present in combinations, are highly predictive of DDT and pyrethroid resistance were detected. Phylogenetic analyses based on the diversity of S989P and V1016G/I mutations were undertaken to assess the phylogeography of these *kdr* mutations.

**Results:**

Genotyping all four *kdr* positions of V410L, S989P, V1016G/I and F1534C revealed that only 16 out of the 56 (28.57%) specimens were homozygous wild type for all *kdr* mutation sites. Six haplotypes including VSVF (0.537), VSVC (0.107), LSVF (0.016), LSIF (0.071), VPGC (0.257) and LPGC (0.011) were detected in this study. For the first time, 11 specimens harbouring the V410L mutation, and 8 samples with V1016I mutation were found. V410L and V1016I were coincided in 8 specimens. Also, six specimens contained 1016G/I double mutation which was not reported before.

**Conclusions:**

The relatively high frequency of these *kdr* mutations in Iranian *Ae. aegypti* indicates a population exhibiting substantial resistance to pyrethroid insecticides, which are used widely in control operations and household formulations. The detection of the 410L/1016I *kdr* mutant haplotype in Iranian *Ae. aegypti* suggests possible convergence of invasive populations from West Africa or Latin America. However, as Iran has very limited maritime/air connections with those African countries, a Latin American origin for the invasive *Ae. aegypti* in Iran is more plausible.

**Graphical abstract:**

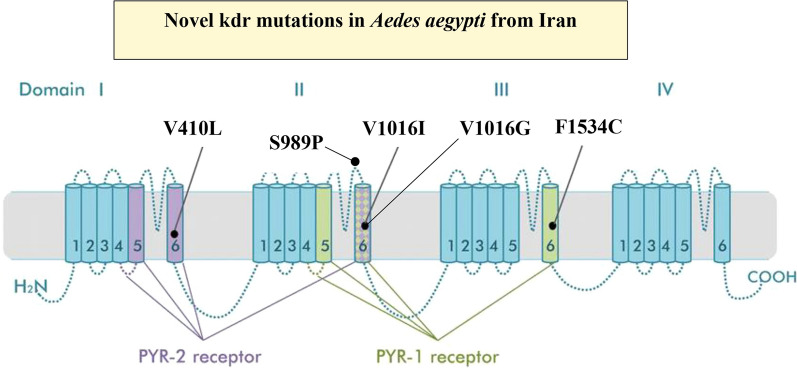

## Background

Almost half of the world population lives in dengue-endemic countries [1]. The global distribution of dengue, chikungunya and Zika is expanding, causing severe disease outbreaks in many urban populations [2]. This increase in disease burden is closely tied to changes in the distribution of the main vector, *Aedes aegypti,* which is in turn largely driven by international trade, travel and climate change [3]. *Aedes aegypti* has invaded the Middle East in recent decades and most of Iran’s neighbouring countries harbour this vector species [4, 5]. Moreover, dengue, the most-important *Aedes*-borne arbovirus, is endemic in Pakistan [6, 7], Afghanistan [8], Saudi Arabia [9, 10], Yemen [11] and to a lesser extent Oman [12]. To date, only few imported cases of dengue have been reported from Iran [13].

Insecticide-based mosquito control is an important strategy for outbreak response and epidemic control. However, insecticide resistance in *Ae. aegypti* is a major threat to effective disease control and may even be a contributing factor to the re-emergence and spread of this species [14]. More than 50 countries have reported resistance to at least one class of insecticide in *Ae. aegypti* [15]. Mutations in the voltage-gated sodium channel (*Vgsc*) typically appear to be the main mechanism of pyrethroid insecticide resistance in *Ae. aegypti* [15, 16]. Multiple *Vgsc* mutations are known in *Ae. aegypti*, of which V410L, S989P, V1016G, V1016I and F1534C appear the most widespread and important for resistance-prediction, especially when combined as double or triple mutant haplotypes [15–17].

Genotyping *Vgsc* gene of different populations of *Ae. aegypti* in Saudi Arabia revealed S989P, V1016G and F1534C mutations causing permethrin and deltamethrin resistance [18, 19]. In Pakistan, India and Sri Lanka, 1534C *kdr* mutation was detected with different frequencies and involved more in DDT and class I than class II pyrethroid insecticide resistance [20–24]. In Thailand, V1016G (homozygous) and F1534C were involved in deltamethrin and permethrin resistance respectively [25–27]. In studies on *Ae. aegypti* from Malaysia and Indonesia, S989P, F1534C and V1016G were detected causing DDT and pyrethroid insecticide resistance [28–32]. V410L was reported for the first time by Haddi et al. in Brazil [33] followed by reports from other countries in the Americas and Africa [34, 35]. *Kdr* alleles and haplotypes differ continentally: 989P and 1016G are common in Asia [28, 32, 36–40]; V410L and 1016I are widespread in the Americas, and 1534C is reported worldwide [16, 17, 41–48]. The V410L and V1016I mutations have recently been detected in African populations of *Ae. aegypti* from Angola, Senegal, Ghana, Cape Verde and Burkina Faso [17, 35, 41, 49–52] and V1016I in Madeira Island (Portugal) in Europe (near Africa) [53]. The *kdr* mutations V410L and V1016I in African populations appear to have originated in the Americas [50].

Different *kdr* mutations exert both qualitatively and quantitatively different effects on the level of insecticide resistance in *Ae. aegypti*. The V1016G mutant alone can cause resistance to pyrethroids, whereas V1016I alone has no effect and also because of fitness cost never occurs in isolation [48, 54]. However, the V1016I + F1534C double-mutant haplotype is more resistant to deltamethrin and permethrin than F1534C alone [42, 48]. Generally, F1534C confers higher resistance to class I pyrethroid insecticides (e.g. permethrin) than to class II pyrethroids (e.g. deltamethrin; alphacypermethrin) but the latter is elevated when in combination with mutants at the 1016 codon [53, 55]. In Latin America, it has been proposed that F1534C first emerged in response to DDT and/or type I pyrethroid use, providing a platform for the subsequent selection of the V1016I and V410L mutants providing a greater level and broader spectrum of pyrethroid resistance [54]. Asian *kdr* variants are also more effective in combinations with the 989P + 1016G haplotype conferring operational resistance to pyrethroid sprays especially when homozygous [56], while addition of the 1534C allele reduces *Vgsc* sensitivity still further [16, 57].

Although *Ae. aegypti* was historically reported from Iran, in the cities of Khorramshahr in 1920 and Bushehr in the early 1950s [58, 59], there has been no recent report of *Ae. aegypti* in the country. Nevertheless, Iran’s ecology and climate are able to support the species [4, 60]. As the species is present in almost all neighbouring countries, reinvasion was expected. *Aedes aegypti* was again detected in Hormozgan Province, southern Iran, in 2020, where the species is now established [13, 61]. Iranian national guidelines for *Ae. aegypti* control recommend environmental management and source reduction followed by insecticide-based interventions [13]. At this early stage of reinvasion, it is challenging to obtain the high numbers of specimens required to perform standard adult susceptibility bioassays, but resistance information is urgently required to implement effective control measures. Given the high predictive value of *kdr* mutations, molecular genotyping studies provide a feasible proxy for likely pyrethroid insecticide resistance patterns that might adversely impact control.

## Methods

### Study area

**Fig. 1:**
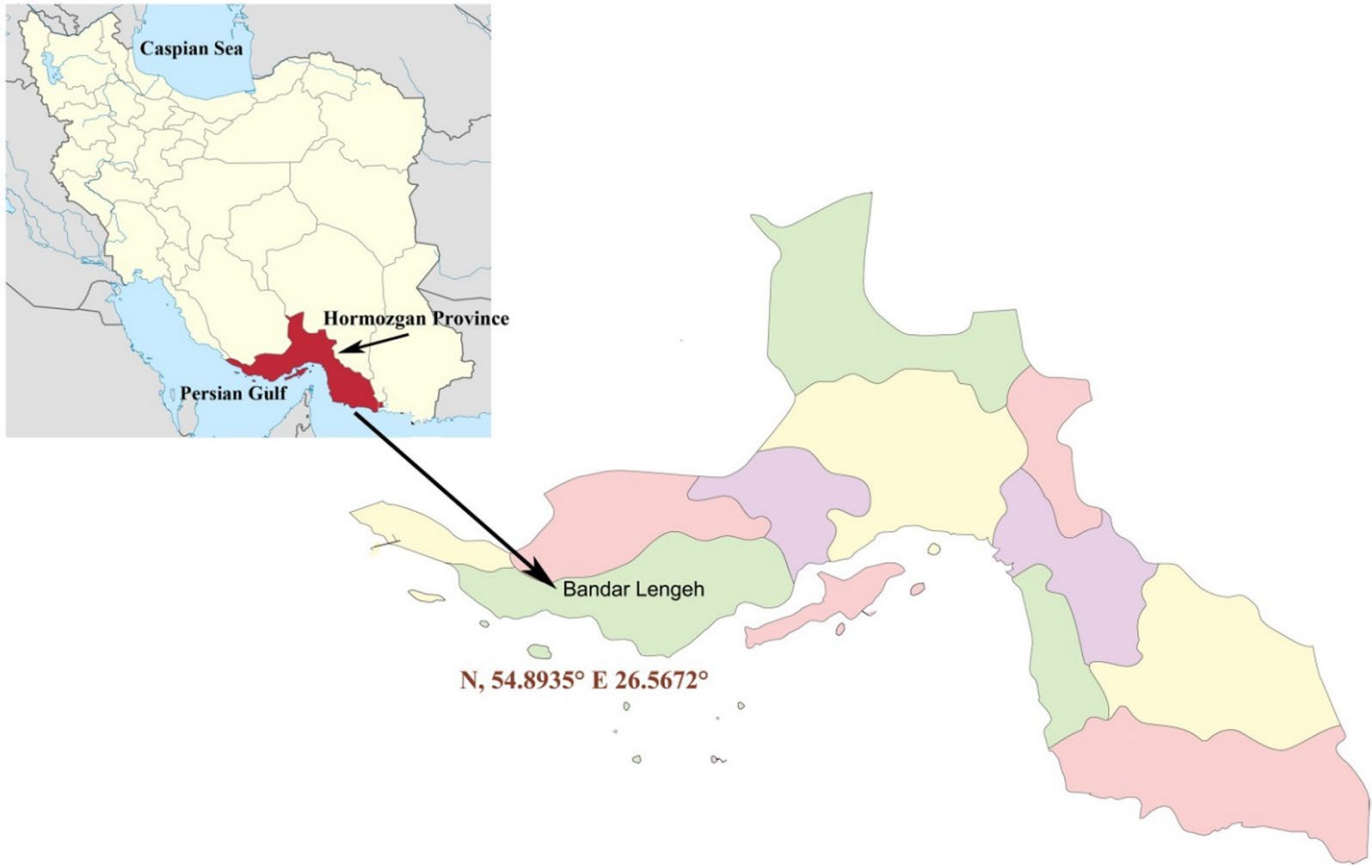
Map of Iran and the study area in the south of the country in Bandar Lengeh

Lengeh is a port city in Hormozgan Province between 26°33′29″N and 54°52′50″ E. The province is located in southern Iran between 53°41'–59°15'E and 25°24'–28°57'N. It is bounded by Fars and Kerman Provinces in the north, Bushehr Province in the west, the Persian Gulf and the Sea of Oman in the south and Sistan and Baluchestan Provinces in the east (Fig. 1). Hormozgan’s climate is warm and humid and the maximum temperature reaches up to 49 °C in summer, while in the winter the minimum temperature can drop to about 5 °C.

### Sample collection

A total of 307 egg, 40 larval, 2 pupal and 5 adult *Ae. aegypti* specimens were collected from January to December 2020 from different areas of Lengeh port by ovitraps, larval (dippers and droppers) and adult (aspirator) collection based on the national guidelines for prevention and control of *Ae. aegypti* and *Aedes albopictus* in Iran [13]. The sampling places were the port as well as inside the city from air conditioner water collection buckets, used tyre piles and broken boats. The coordinates of the exact sampling sites are given in Appendix 1. Immature stages were reared to adults in an insectary with standard conditions of 28 ± 2 °C and 75 ± 5% relative humidity and fed with TetraMin® flakes. Altogether 56 adult *Ae. aegypti* specimens were available for molecular analysis.

### DNA isolation

DNA from individual mosquitoes was extracted using the Livak buffer extraction method [62] with some modifications [63]. In brief, individual mosquitoes were homogenized in 100 μl pre-heated (65 °C) Livak buffer in 1.5-ml Eppendorf tubes using a plastic pestle. Homogenates were incubated at 65 °C for 30 min. Potassium acetate was added to each tube to a final concentration of 1 M before incubating the mixture on ice for 30 min. The tubes were centrifuged at 12,000*g* for 15 min at 4 °C. Supernatants were transferred to clean tubes and mixed with 200 μl ice-cold ethanol, followed by centrifugation at 12,000*g* for 15 min at 4 °C. Pellets were rinsed in 100 μl 70% ice-cold ethanol, spun at 12,000*g* for 5 min at 4 °C and re-suspended in 50 μl pre-heated Tris–EDTA (TE) buffer or nuclease-free water.

### Primers

**Fig. 2:**
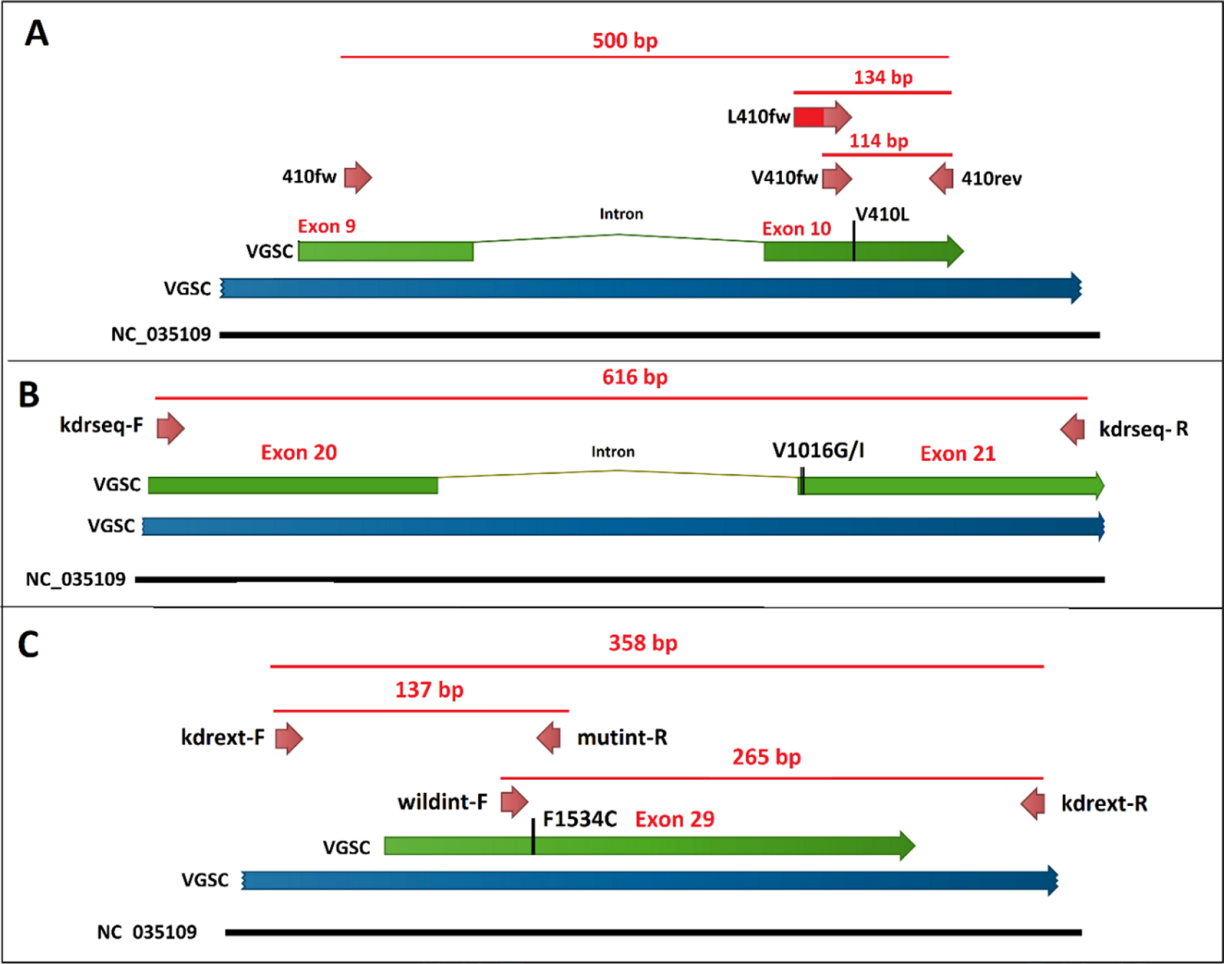
Schematic diagram of the stretch of the *Vgsc* gene of *Aedes aegypti* depicting where the primers sit in three different PCR reactions for genotyping V410L, S989P, V1016G/I and F1534C *kdr* mutations. A: PCR1, B: PCR2 and C: PCR3

Three different sets of primers were designed/used to amplify three separate fragments of the IS6, IIS6 and IIIS6 of the *Vgsc* gene of *Ae. aegypti* in PCR reactions (Table 1). V410fw, L410fm and 410rev primers were used in an allele-specific PCR followed by melting curve analysis to genotype the V410L position [55]. In PCR2, using primers kdrseq-F and kdrseq-R, a flanking region of 633 bp in domain II of the *Vgsc* gene was amplified followed by sequencing in both directions to detect the S989P and V1016G/I mutations. Two external primers, kdrext-F and kdrext-R, as well as two specific primers, wildint-F and mutant-R, were used in an allele-specific PCR (PCR3) to genotype the F1534C position of the *Vgsc* gene of *Ae. aegypti* according to Harris et al. with some modifications [64]. Some of the PCR1 and PCR3 products were also sequenced using the external primer pairs (Table 1) to ensure that the genotyping techniques worked well. A schematic diagram showing the stretch of the IS6, IIS5-6 and IIIS6 of the *Vgsc* gene where the primers bind is given in Fig. 2.

**Table 1:** Primers used in different PCR of the *Vgsc* gene of *Aedes aegypti* from Iran

Primer	Purpose	Sequence (5′ to 3′)	Length	Refs.
V410fw	Allele-specific PCR, melting curve analysis, wild genotype (IS6)	GCGGGCAGGGCGGCGGGGGCGGGGCCA TCTTCTTGGGTTCGTTCTACCGTG	51	[55]
L410fw	Allele-specific PCR, melting curve analysis, mutant genotype (IS6)	GCGGGCATCTTCTTGGGTTCGTTCTACCATT	31	[55]
410rev	Allele-specific PCR, melting curve analysis, reverse primer (IS6)	TTCTTCCTCGGCGGCCTCTT	20	[55]
410fw	Sequencing V410L position	GATAATCCAAATTACGGGTATAC	23	[55]
kdrseq-F	PCR and sequencing (IIS6)	AGACAATGTGGATCGCTTC	19	Investigator-designed
kdrseq-R	PCR and sequencing (IIS6)	ACGCAATCTGGCTTGTTA	18	Investigator-designed
kdrext-F	Allele-specific PCR (IIIS6)	CGAACTTGTTACGAATGATCTGCTTAC	27	[64]
kdrext-R	Allele-specific PCR (IIIS6)	TGAGAATAGCGAGATTAGGAAGGAA	25	[64]
wildint-F	Allele-specific PCR (IIIS6)	CCTCTACTTTGTGTTCTTCATCATTTG	27	[64]
mutint-R	Allele-specific PCR (IIIS6)	GCGTGAAGAACGACCTGA	18	[64]

### PCR protocols

In the PCR1, the reaction mixture (25 μl) contained 10 µl RealQ Plus 2 × Master Mix Green (Ampliqon, Denmark), 0.4 µM of each primer and 100 ng of the extracted DNA. The reactions were run on a StepOnePlus™ Real-Time PCR System (Applied Biosystems, USA) by the following protocol: an initial denaturation and hot-start enzyme activation at 95 °C for 10 min, then 37 cycles at 94 °C for 30 s, 60 °C for 30 s and 72 °C for 30 s, and a final extension step at 72 °C for 7 min. Two unequal-sized PCR products produced by the wild-type and mutant alleles were differentiated by melt curve analysis at 65 °C to 95 °C in 0.1 °C increment steps on the device [55]. PCR2 was performed in a reaction mixture (25 μl) that contained 1 × buffer, 1.5 mM of MgCl2, 200 μM of each dNTP, 0.5 μM of each kdrext-F and kdrext-R primer and 0.625 unit of High-Fidelity DNA Polymerase (Ampliqon, Denmark). The PCR protocol was: an initial denaturation at 95 °C for 2 min, followed by 35 cycles at 95 °C for 30 s, 60 °C for 30 s and 72 °C for 45 s, and a final extension step at 72 °C for 7 min. The products of the PCR 2 were run on a 2% agarose gel and the bands were cut and the DNA extracted using a PCR purification kit (Takapozist, Iran); they were sequenced in both directions (Macrogen Inc., South Korea) using BigDye (Applied Biosystem Chemistry). PCR3 protocol was similar to PCR2 except that all four primers were used in each reaction and the products were run on 2% agarose gel to observe different banding patterns of 137 bp (F1534C: mutant), 265 bp (F1534: wild type) and 358 bp (control).

### Data analysis

Melting curve analysis of PCR1 was performed using StepOnePlus software (Applied Biosystems, USA) for V410L genotyping. All the sequencing data of the PCR2 products were analysed using CLC Genomics Workbench software v20.0.04 (QIAGEN, Germany) and the mutations at the S989P and V1016G/I positions were identified by aligning them with the reference sequences (gene ID: 5567355). Three different nucleotide sequences containing S909P, V1016G, V1016I and 1016G/I were used as queries to search for similar sequences in GenBank using nucleotide blast (https://blast.ncbi.nlm.nih.gov/Blast.cgi). All the existing sequences containing mutations in the S989P and V1016G/I positions in the database were downloaded and used for the phylogenetic analysis using CLC Genomics Workbench. For PCR3, banding pattern differences on 2% agarose gel were used to analyse the allele-specific PCR products to genotype F1534C *kdr* mutation. The phylogenetic tree was constructed using the UPGMA method with 1000 bootstrap replicates by CLC Genomics Workbench software v20.0.04. Chi-square statistical test was used to assess the correlation between different genotypes in a SPSS v22 software at the significance level of 5%.

## Results

**Fig. 3:**
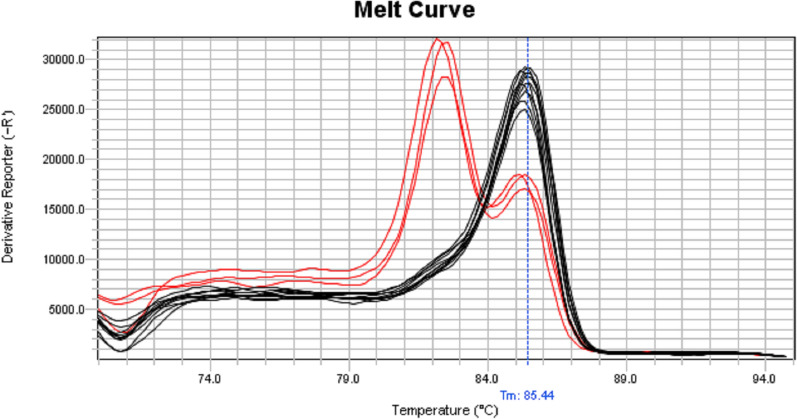
Allele-specific melting-curve real-time PCR of the IS6 of the *Vgsc* gene of *Aedes aegypti* from Iran. V410L heterozygotes (in red) produced two peaks at about 82.5 °C (410L) and 85.5 °C (V410). V410-susceptible homozygote produced a single peak at about 85.5 °C (in black)

**Fig. 4:**
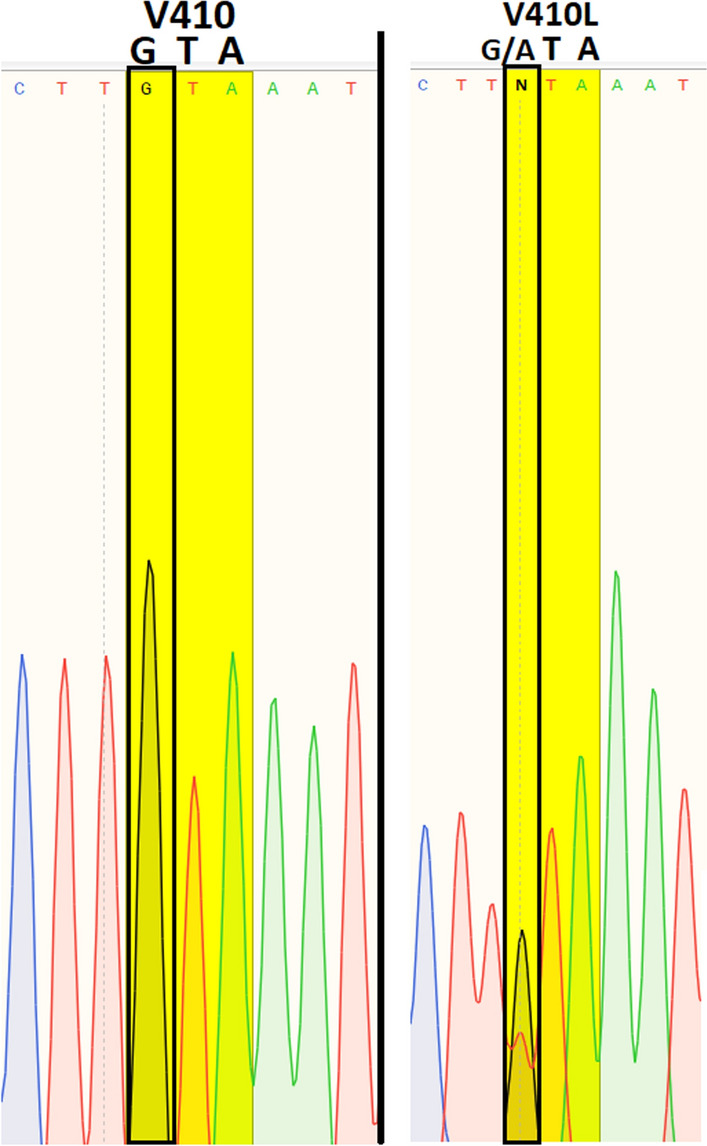
Sequence of a stretch of *Vgsc* gene of *Aedes aegypti* from Iran showing the V410L mutation (GG: homozygote wild, GA: heterozygote). GTA and ATA genetic codes for valine and leucine respectively

**Fig. 5:**
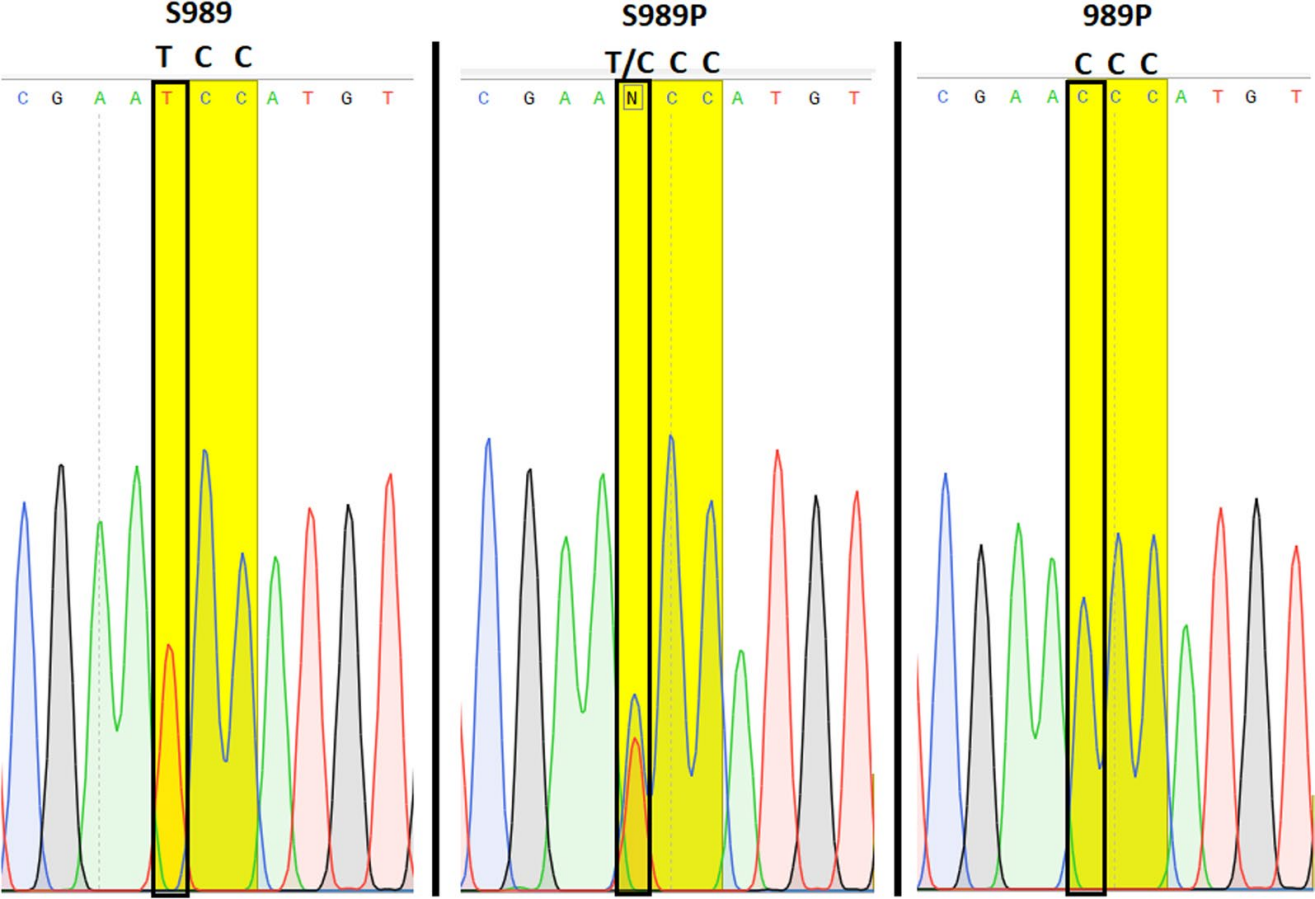
Sequence of a stretch of *Vgsc* gene of *Aedes aegypti* from Iran showing the S989P mutation (CC: homozygote mutant, CT: heterozygote and TT: wild type). TCC and CCC genetic codes for serine and proline respectively

**Fig. 6:**
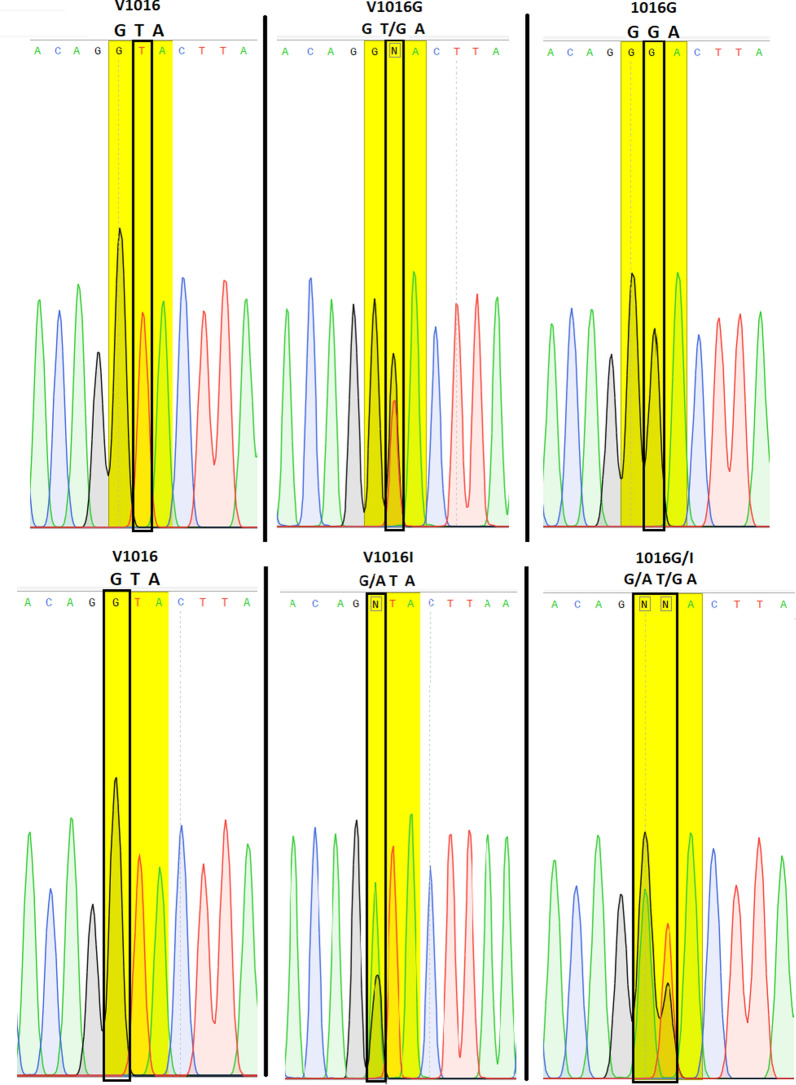
Sequence of a stretch of *Vgsc* gene of *Aedes aegypti* from Iran showing the V1016G/I mutations (GG: homozygote mutant, GT: heterozygote and TT: wild type; GA: heterozygote 1016I). GTA, GGA and ATA genetic codes for valine, glycine and isoleucine respectively

**Fig. 7:**
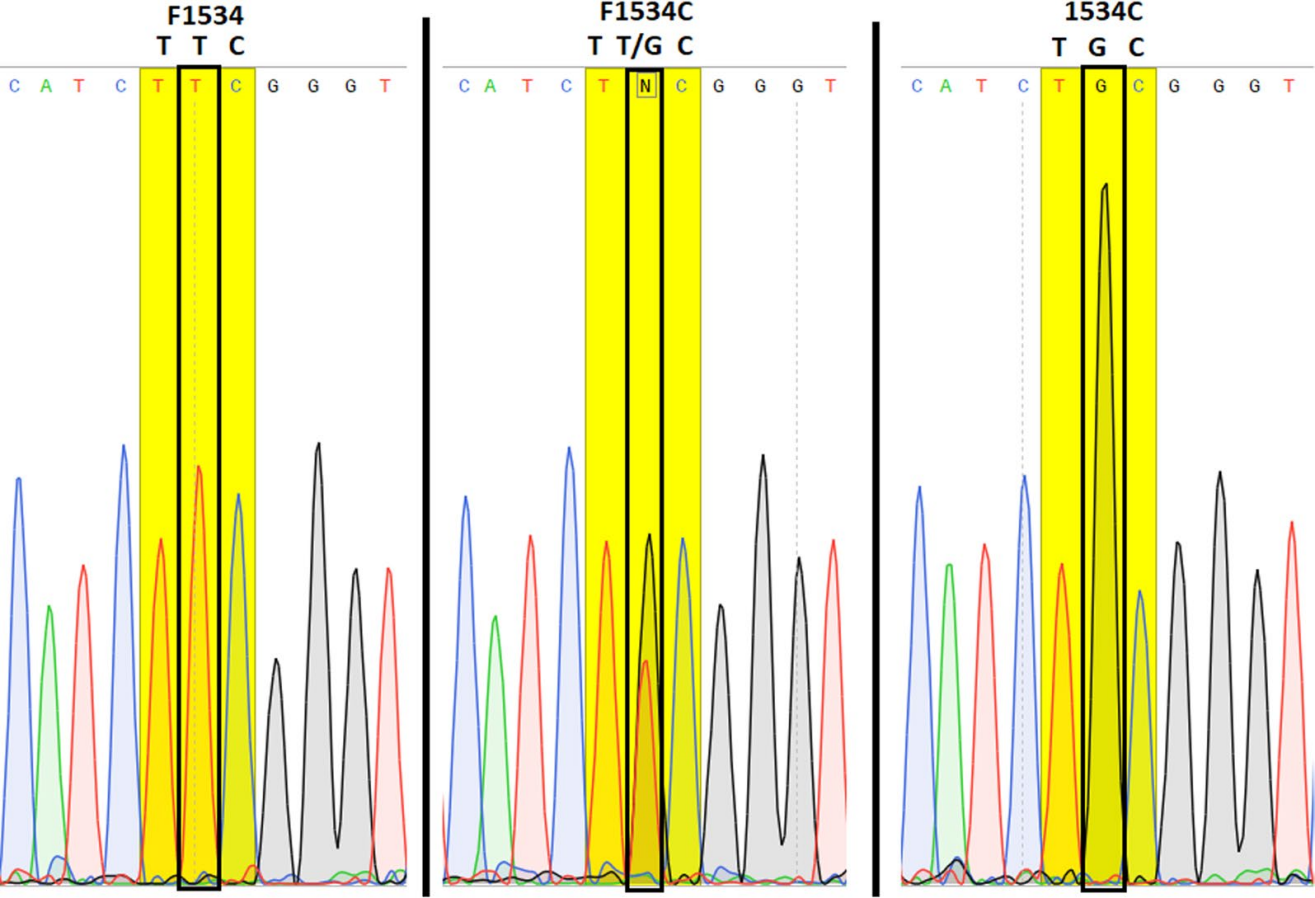
Sequence of a stretch of *Vgsc* gene of *Aedes aegypti* from Iran showing the F1534C mutation (GG: homozygote mutant, GT: heterozygote and TT: wild type). TTC and TGC genetic codes for phenylalanine and cysteine respectively

**Fig. 8:**
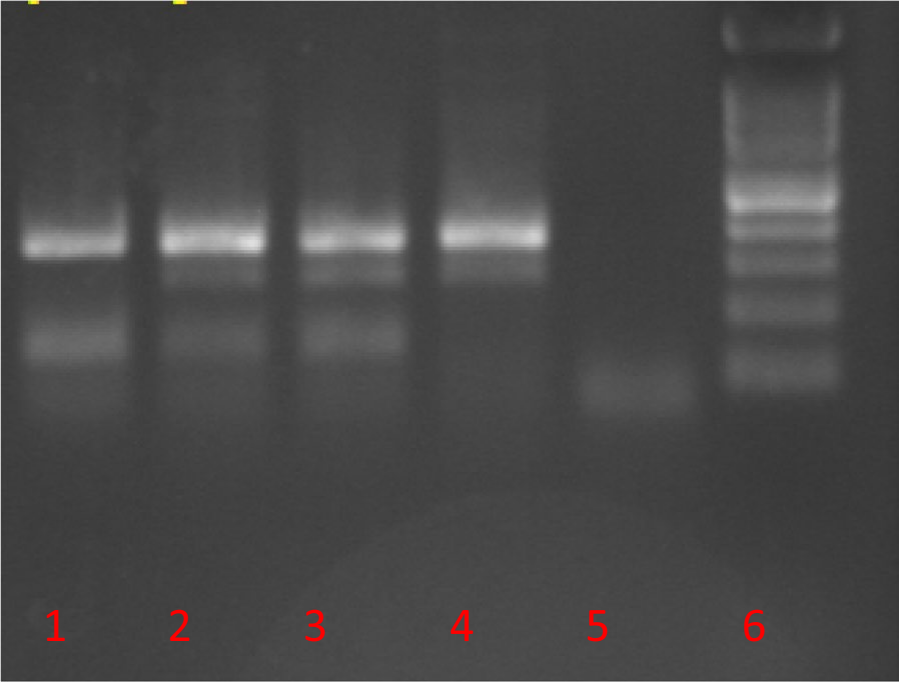
Agarose gel electrophoresis of allele specific PCR3 product showing different banding patterns for the F1534C mutation in *Vgsc* gene of *Aedes aegypti* from Iran. Lane 1: homozygote mutant; lanes 2 and 3: heterozygote; lane 4: homozygote wild; lane 5: negative control and lane 6: DNA ladder

Molecular analyses were successfully performed on all 56 specimens of *Ae. aegypti* collected from Lengeh Port City. Allele-specific PCR followed by melting curve analysis for genotyping the V410L position of the *Vgsc* gene of *Ae. aegypti* from Iran revealed peaks with different melting points (Fig. 3). V410L heterozygotes (in red) produced two peaks at 82.5 °C (410L) and 85.5 °C (V410). V410 susceptible homozygote produced a single peak at 85.5 °C (in black). Some of the PCR products of the V410L position were sequenced to assure the sensitivity of the melting curve analyses (Fig. 4). PCR followed by sequencing (PCR2) and allele-specific PCR (PCR3) revealed several *kdr* mutations in the *Vgsc* gene of the mosquitoes including S989P, V1016G, V1016I, 1016G/I and F1534C. Chromatograms of the mutations S989P, V1016G, V1016I and 1016G/I are shown in Figs. 5, 6, 7. Banding patterns showing the detection of the F1534C mutation are depicted in Fig. 8.

Based on the *kdr* genotyping results of 56 Iranian *Ae. aegypti*, 16 (28.57%) specimens were homozygote wild type at all positions. The frequencies of the genotypes and haplotypes are summarized in the Appendix 2 and Tables 2 and 3. At the V410L position, 11 specimens (19.6%) were heterozygote and no individual homozygous for 410L was found. The frequency of S989, S989P and 989P alleles was 50% (28 specimens), 46.4% (26) and 3.6% (2) respectively. At the V1016G/I position, 26 (46%), 20 (36%), 2 (3.6%), 2 (3.6) and 6 (11%) specimens were wild type, heterozygous for 1016G, homozygous for 1016G, heterozygous for 1016I and heterozygous for 1016G/I respectively. At the F1534C position, 19 (34%), 32 (57%) and 5 (9%) specimens were homozygote wild type, heterozygote and homozygote mutant respectively. All V410L specimens were also heterozygous for V1016I or 1016G/I mutations with statistically significant association (χ^2^ = 0.001). Six haplotypes with different frequencies including VSVF (0.537), VSVC (0.107), LSVF (0.016), LSIF (0.071), VPGC (0.257) and LPGC (0.011) were detected in this study. About half of the haplotypes contain at least one *kdr* mutation. A haplotype containing 1016I mutation has never been seen with S989P. The results of genotyping *kdr* mutations at different domains of the *Vgsc* gene of *Ae. aegypti* from Iran indicate relatively strong DDT and pyrethroid insecticide resistance.

**Table 2:** List and frequency of haplotypes and genotypes of *Aedes aegypti* from Iran

Haplotype	Freq	V410L_A410T	S989P_A_989_T	V1016G/I	F1534C_A1534T	E2021intronAB_AT
VSVFB	0.519	V	S	V	F	B
V**PGC**A	0.23	V	P	G	C	A
VSV**C**B	0.098	V	S	V	C	B
**L**S**I**FA	0.071	L	S	I	F	A
V**PGC**B	0.027	V	P	G	C	B
VSVFA	0.018	V	S	V	F	A
**L**SVFB	0.016	L	S	V	F	B
L**PGC**A	0.011	L	P	G	C	A
VSV**C**A	0.009	V	S	V	C	A

**Table 3:** List and frequency of haplotypes and the number of *kdr* alleles in *Aedes aegypti* from Iran

Haplotype	Freq	*kdr* alleles
VSVF	0.537	0
VSV**C**	0.107	1
**L**SVF	0.016	1
**L**S**I**F	0.071	2
V**PGC**	0.257	3
**LPGC**	0.011	4

In this study, eight specimens were detected harbouring 1016I mutation. All specimens heterozygous for 1016I were also heterozygous for V410L. The detection of V410L and V1016I mutations in *Vgsc* of *Ae. aegypti* from Iran is the first such report from Asia. Six of eight individuals heterozygous for mutations at the 1016 position have a double-mutation 1016G/1016I genotype. This genotype has not been reported before. The sequencing results of the flanking region of the *Vgsc* gene of Iranian *Ae. aegypti* spanning 989 and 1016 positions (PCR2) are available in GenBank under the accession numbers OK236520, MZ773476 and MZ773475.

**Fig. 9:**
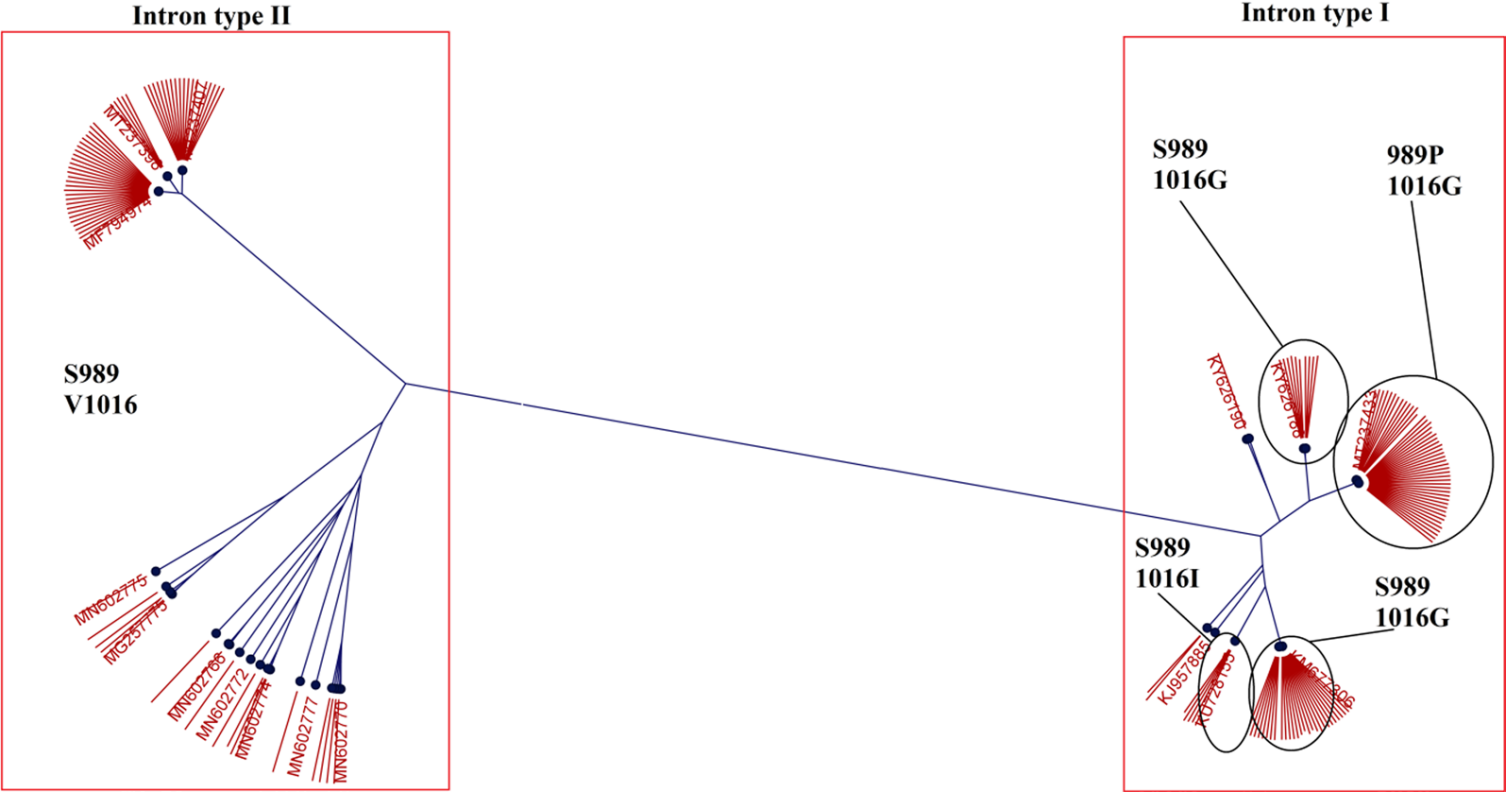
Phylogenetic tree of the *kdr* mutations at S989P and V1016G/I positions of the *Vgsc* gene of *Aedes aegypti* from Iran

The sequencing results of the IIS6 section of the *Vgsc* gene of Iranian *Ae. aegypti* also revealed polymorphism in the intron between exons 20 and 21 including intron type A (250 bp) and intron type B (234 bp). The frequency of intron A, B and AB genotypes was 14.3%, 46.4% and 39.3% respectively (Annex 2). In this study, all six 1016G/I individuals were linked with type A intron, an association that is statistically significant (χ2 = 0.001). Intron B was observed in all specimens homozygous wild type for all *kdr* positions tested (χ2  = 0.001). Phylogenetic analysis was performed on the *kdr* mutations at S989P and V1016G/I positions of the *Vgsc* gene. The result of the analysis in the form of the phylogenetic tree is presented in Fig. 9. All sequences harbouring the 989P and 1016G/I *kdr* mutations fell into the branch containing intron type A. The sequence of 1016I *kdr* mutation of the Iranian population of *Ae. aegypti* fell in the clade of sequences available in GenBank all from the Americas.

## Discussion

Five *kdr* mutations including V410L, S989P, V1016G, V1016I and F1534C were detected in Iranian *Ae. aegypti* with different frequencies. For an invasive species that has not been under severe and long enough local selection pressure to generate in situ *kdr* mutations [50], the overall frequencies of the *kdr* mutations are considerable, raising concern about the effectiveness of pyrethroid insecticide-based interventions. The relatively high frequency of the S989P + V1016G/I haplotype on its own or in combination with V410L and F1534C confers high pyrethroid insecticide resistance [15, 17], which may lead to operational failure of these insecticides against *Ae. aegypti* under field conditions. A combination of 989P + 1016G confers about fivefold deltamethrin resistance compared with 1016G alone, and that combination of triple *kdr* mutation 989P + 1016G/I + 1534C exerts even stronger resistance to pyrethroids (50-fold) in *Ae. aegypti*. Space spraying when the population is triple heterozygous *kdr* mutant S989P + V1016G/I + F1534C may also fail [26]. These findings are also backed by studies on *Ae. aegypti* from other countries [39, 41, 50, 65]. V410L is also a potent *kdr* mutation which leads to pyrethroid insecticide resistance on its own and even more so when combined with other *kdr* mutations [33, 34].

The fact that about 70% of the specimens of *Ae. aegypti* from Iran were either heterozygous or homozygous for all four *kdr* mutations studied, is a cause for concern regarding the control of *Ae. aegypti* using pyrethroid insecticides in Iran. This part of the results corroborates with similar studies on *Ae. aegypti* in the world where about the same frequencies of *kdr* mutations caused high pyrethroid insecticides resistance [17, 21, 66–68]. The frequency of the F1534C mutation was higher than other *kdr* mutations in our study. Considering the correlation between this *kdr* mutation and DDT/permethrin resistance, and also noting that the frequencies of F1534C and 1534C were 57% and 9% in this study, it can be concluded that the resistance to permethrin should be high in Iranian *Ae. aegypti* [16, 17, 21, 24, 53, 69]. On the other hand, apart from 1016G, which is pivotal in pyrethroid resistance on its own [32], haplotypes harbouring triple mutations of S989P, V1016G/I and F1534C showed a frequency of 45% with implication in pyrethroid insecticide resistance. In addition, V410L with a frequency of about 20% exacerbates the pyrethroid insecticide resistance levels [16, 17, 28, 30, 33, 70, 71]. In other words, only < 30% of the specimens were homozygote wild type for all *kdr* positions studied, which means that the development of pyrethroid insecticide resistance started long ago in Iranian *Ae. aegypti*. As the species has recently invaded Iran and not been under long local selection pressure, this relatively high frequency of *kdr* mutations reveals that the evolution of resistance to pyrethroid insecticides might have been started in the ultimate country of origin of the mosquito. Not only the frequency of the F1534C mutation is higher than for the rest of the mutations, but about 9% of the specimens were homozygous for 1534C. These statistics indicate that the development of the F1534C mutation either started earlier or at a faster pace than the rest of the mutations [54]. In India, a new T1520I mutation was recently identified along with F1534C, which might have an enhancing effect on F1534C regarding protection against permethrin [22, 54, 72].

Six different haplotypes, VSVF, VSVC, LSVF, LSIF, VPGC and LPGC, were detected in this study; except for one (VSVF), the rest harbour at least one *kdr* mutation. The sum of the frequency of the haplotypes containing at least one *kdr* mutation is about 50%, which is high enough to exert a rather strong pyrethroid insecticide resistance in Iranian *Ae. aegypti*. Notably, the LPGC haplotype, which was detected in this study with rather high frequency, has not been reported in any *kdr* studies worldwide [16]. Different haplotypes confer differentially to pyrethroid insecticide resistance. Haplotypes harbouring V410L mutation either alone or in combination with the F1534C were strongly associated with increased resistance to type I and especially II pyrethroids. The order of haplotypes exerting permethrin resistance is 410L + 1534C > 410L > 1534C, and concerning deltamethrin resistance, the order is 410L = 410L + 1534C >  > 1534C [33].

For the first time in Asia, the of V410L and V1016I mutations in *Vgsc* gene were detected in *Ae. aegypti* from Iran. Although V410L and V1016I mutations have been reported in multiple African countries [17, 41, 49–53], Iran has limited or no maritime/air connections with those West African countries. Contrarily, in the recent decades, the country has expanded its trade and travel to the Americas. As most of the populations harbouring these mutations have their origin in the Americas, it can cautiously be concluded that the newly established Iranian population of *Ae. aegypti* might have the same origin. Many believe that V410L and V1016I mutations detected in Africa are due to mosquitoes brought back from Latin America just like the triangular Atlantic trips between Europe, Africa, South America and back to Europe that brought *Ae. aegypti* to Europe in the 1800s [22, 72]. Nonetheless, an African origin for Iranian *Ae. aegypti* cannot be confidently ruled out pending further molecular and population genetics studies. Iran and Pakistan have a common border of about 900 km; noting the presence of *Ae. aegypti* throughout Pakistan makes this country one of the prime places from which the mosquito would be expected to enter Iran. In recent studies in Pakistan, no *kdr* mutations were identified in sequencing the IS6 and IIS6 regions of the *Vgsc* gene of *Ae. aegypti* specimens [20, 73], whereas in our study, a relatively high frequency of mutation in IS6 and IIS6 has been found, results that probably rule out the Pakistani origin of Iranian *Ae. aegypti*. In a study using COI sequencing, the origin of Iranian *Ae. aegypti* was claimed to be Kenya or Panama [61]. However, the result is not well supported by a rather short length (310-bp) *COI* gene amplified from a single mosquito specimen [61].

Another very important observation of this study is that six out of eight heterozygote individuals for mutation at the 1016 position have a double-mutation 1016G/1016I genotype. This genotype has not been reported before. As V1016I has so far been reported mainly from the Americas and V1016G mainly from Asia, this discovery may indicate that the population of *Ae. aegypti* from Bandar Lengeh is the result of two different introductions, one from Asia and one from the Americas (or less likely from Africa), and the reproduction of the two populations yielded the hybrid genotype form of 1016G/1016I. As the discovery of V410L and V1016I mutations in our study is new to Asia and also because the 1016G/I mutation has not been reported in any *Ae. aegypti* populations worldwide, more molecular and population genetic studies are required to elucidate their formation.

Apart from the merit of the detection of V410L and V1016I *kdr* mutations in indicating the origin of the Iranian *Ae. aegypti*, phylogenetic analysis of the *kdr* mutations at S989P and V1016G/I positions obtained in our study may also serve the purpose. First, based on the available sequences in GenBank, all the populations with those *kdr* mutations have type A intron regardless of the geographical origin [17, 39]; second, the clade harbouring 1016I sequences is mostly from Latin America.

## Conclusions

The results of this molecular study revealed relatively high frequencies of *kdr* mutations V410L, S989P, V1016G/I and F1534C in the newly invaded Iranian population of *Ae. aegypti*. The frequencies as well as the correlation/co-occurrence between these mutations are indicative of a relatively high impact on the effectiveness of pyrethroid-based chemical control interventions against *Ae. aegypti* in Iran. Therefore, emphasis is as always put on non-chemical interventions like environmental management and source reduction, and where chemical control measures are inevitable, non-pyrethroid insecticides or two-in-one (pyrethroid + non-pyrethroid) formulations are strongly recommended in line with insecticide resistance management principals. Further investigation of the dimensions and operational impact of insecticide resistance in *Ae. aegypti* from Iran including bioassays for determination of the frequency and intensity of resistance to different insecticides, biochemical and more in-depth molecular studies are recommended. The discovery of 11 specimens with V410L and 8 individuals with 1016I mutations that have so far not been reported in Asia, but are common in the Americas, may provide indications that the Iranian *Ae. aegypti* might have originated from the Americas; more population genetics studies on a higher number of specimens are recommended to further investigate this hypothesis.

## Data Availability

The data supporting the findings of the study must be available within the article and/or its supplementary materials, or deposited in a publicly available database.

## Appendices

### Appendix 1

**Table:** The coordinates of the sampling places and the number of eggs, larvae, pupae and adults collected during 2020

	Latitude	Longitude	Egg	Larva	Pupa	Adult
1	26° 33′ 53.07"	54° 53′ 29.57"	0	2	0	0
2	26° 32′ 57.29"	54° 53′ 00.67"	82	0	0	4
3	26° 35′ 35.48"	54° 55′ 59.00"	0	0	0	1
4	26° 32′ 39.29"	54° 52′ 31.16"	25	4	2	0
5	26° 33′ 3.39"	54° 53′ 10.28"	135	3	0	0
6	26° 32′ 36.58"	54° 52′ 27.19"	3	6	0	0
7	26° 35′ 20.41"	54° 56′ 11.04"	33	4	0	0
8	26° 33′ 06.01"	54° 51′ 59.1"	0	4	0	0
9	26° 34′ 21.10"	54° 53′ 33.35"	0	5	0	0
10	26° 32′ 46.65"	54° 52′ 40.74"	29	12	0	0
Sum			307	40	2	5

### Appendix 2

**Table:** List of *kdr* mutations and genotypes in association with the intron type in *Vgsc* gene of *Aedes aegypti* from Iran

No.	Specimen code	V410L	F989P	V1016G	V1016I	F1534C	Intron	Genotype
1	1	W	W	W	–	W	B	V410/S989/V1016/F1534
2	7	W	W	W	–	W	B	V410/S989/V1016/F1534
3	L4	W	W	W	–	W	B	V410/S989/V1016/F1534
4	L6	W	W	W	–	W	B	V410/S989/V1016/F1534
5	L9	W	W	W	–	W	B	V410/S989/V1016/F1534
6	L26	W	W	W	–	W	B	V410/S989/V1016/F1534
7	L29	W	W	W	–	W	B	V410/S989/V1016/F1534
8	L30	W	W	W	–	W	B	V410/S989/V1016/F1534
9	L34	W	W	W	–	W	B	V410/S989/V1016/F1534
10	L35	W	W	W	–	W	B	V410/S989/V1016/F1534
11	L36	W	W	W	–	W	B	V410/S989/V1016/F1534
12	L38	W	W	W	–	W	B	V410/S989/V1016/F1534
13	L45	W	W	W	–	W	B	V410/S989/V1016/F1534
14	L47	W	W	W	–	W	B	V410/S989/V1016/F1534
15	L53	W	W	W	–	W	B	V410/S989/V1016/F1534
16	L55	W	W	W	–	W	B	V410/S989/V1016/F1534
17	L18	W	W	W	–	H	B	V410/S989/V1016/F1534C
18	L19	W	W	W	–	H	B	V410/S989/V1016/F1534C
19	L1	W	W	W	–	H	B	V410/S989/V1016/F1534C
20	L5	W	W	W	–	H	B	V410/S989/V1016/F1534C
21	L42	W	W	W	–	H	B	V410/S989/V1016/F1534C
22	L49	W	W	W	–	H	B	V410/S989/V1016/F1534C
23	L52	W	W	W	–	H	B	V410/S989/V1016/F1534C
24	L27	W	W	W	–	M	B	V410/S989/V1016/1534C
25	L54	W	W	W	–	M	B	V410/S989/V1016/1534C
26	L28	W	H	H	–	H	AB	V410/S989P/V1016G/F1534C
27	2	W	H	H	–	H	AB	V410/S989P/V1016G/F1534C
28	8	W	H	H	–	H	AB	V410/S989P/V1016G/F1534C
29	3	W	H	H	–	H	AB	V410/S989P/V1016G/F1534C
30	L31	W	H	H	–	H	AB	V410/S989P/V1016G/F1534C
31	L32	W	H	H	–	H	AB	V410/S989P/V1016G/F1534C
32	L33	W	H	H	–	H	AB	V410/S989P/V1016G/F1534C
33	L40	W	H	H	–	H	AB	V410/S989P/V1016G/F1534C
34	L14	W	H	H	–	H	AB	V410/S989P/V1016G/F1534C
35	L43	W	H	H	–	H	AB	V410/S989P/V1016G/F1534C
36	L44	W	H	H	–	H	AB	V410/S989P/V1016G/F1534C
37	5	W	H	H	–	H	AB	V410/S989P/V1016G/F1534C
38	L15	W	H	H	–	H	AB	V410/S989P/V1016G/F1534C
39	L51	W	H	H	–	H	AB	V410/S989P/V1016G/F1534C
40	L10	W	H	H	–	H	AB	V410/S989P/V1016G/F1534C
41	L11	W	H	H	–	H	AB	V410/S989P/V1016G/F1534C
42	L56	W	H	H	–	H	AB	V410/S989P/V1016G/F1534C
43	L13	W	M	M	–	M	A	V410/989P/1016G/1534C
44	L46	W	M	M	–	M	A	V410/989P/1016G/1534C
45	6	W	H	H	–	M	AB	V410/S989P/V1016G/1534C
46	4	H	W	W	–	W	B	V410L/S989/V1016/F1354
47	L48	H	W	–	H	W	AB	V410L/S989/V1016I/F1534
48	L7	H	W	–	H	W	AB	V410L/S989/V1016I/F1534
49	L41	H	H	M	M	H	A	V410L/S989P/V1016G&I/F1534C
50	L37	H	H	M	M	H	A	V410L/S989P/V1016G&I/F1534C
51	L3	H	H	M	M	H	A	V410L/S989P/V1016G&I/F1534C
52	L2	H	H	M	M	H	A	V410L/S989P/V1016G&I/F1534C
53	L50	H	H	M	M	H	A	V410L/S989P/V1016G&I/F1534C
54	L39	H	H	M	M	H	A	V410L/S989P/V1016G&I/F1534C
55	L16	H	H	H	–	H	AB	V410L/S989P/V1016G/F1534C
56	L17	H	H	H	–	H	AB	V410L/S989P/V1016G/F1534C

W: wild; H: heterozygous; M: mutant
