# Correction:* Kdr* genotyping and the first report of V410L and V1016I* kdr* mutations in voltage-gated sodium channel gene in* Aedes aegypti* (Diptera: Culicidae) from Iran

**DOI:** 10.1186/s13071-026-07269-5

**Published:** 2026-03-30

**Authors:** Ahmadali Enayati, Reza Valadan, Mahboobeh Bagherzadeh, Mohammad Cheraghpour, Seyed Hassan Nikookar, Mahmoud Fazeli-Dinan, Nasibeh Hosseini-Vasoukolaei, Farzaneh Sahraei Rostami, Razieh Shabani Kordshouli, Ahmad Raeisi, Fatemeh Nikpour, Abdolreza Mirolyaei, Fatemeh Bagheri, Mohammad Mehdi Sedaghat, Morteza Zaim, David Weetman, Janet Hemigway

**Affiliations:** 1 Department of Medical Entomology and Vector Control, School of Public Health and Health Sciences Research Center, Mazandaran University of Medical Sciences, Sari, Iran; 2 Department of Immunology and Molecular and Cellular Biology Research Center, Mazandaran University of Medical Sciences, Sari, Iran; 3 Department of Medical Entomology and Vector Control, School of Public Health, Student Research Center, Mazandaran University of Medical Sciences, Sari, Iran; 4 Health Sciences Research Center, Department of Medical Entomology and Vector Control, School of Public Health, Mazandaran University of Medical Sciences, Sari, Iran; 5 Department of Medical Entomology and Vector Control, School of Public Health, Mazandaran University of Medical Sciences, Sari, Iran; 6 Vector Borne Diseases Control Department, Iran CDC, Ministry of Health and Medical Education, Tehran, Iran; 7 Department of Medical Parasitology & Mycology, Tehran University of Medical Sciences, Tehran, Iran; 8 Department of Environmental Chemical Pollutants and Pesticides, Institute for Environmental Research, Tehran University of Medical Sciences, Tehran, Iran; 9 Hormozgan Provincial Health Center, Department of Communicable Diseases Control, Hormozgan University of Medical Sciences, Bandar Abbas, Iran; 10 Department of Medical Entomology and Vector Control, Tehran University of Medical Sciences, Tehran, Iran; 11 Vector Biology Department, Liverpool School of Tropical Medicine, Liverpool, UK

**Correction: Parasites & Vectors (2024) 17:34** 10.1186/s13071-024-06123-w

In the original article [1], it has come to our attention that the graphical abstract included in the publication was adapted from Fig. 1 of the paper by Saavedra-Rodriguez et al. [2], without providing explicit acknowledgment or credit to the original source in the graphical abstract. Although the work by Saavedra-Rodriguez et al. was cited in our paper seven times, it was not cited within the graphical abstract and while this omission was clearly an oversight, not a deliberate action, we wish to clarify that the graphical abstract was adapted from the figure under a Creative Commons Attribution 4.0 (CC BY 4.0) license. In accordance with the license requirements, proper attribution has now been provided to the original author(s) as follows:

Saavedra-Rodriguez K, Maloof FV, Campbell CL, Garcia-Rejon J, Lenhart A, Penilla P, et al. Parallel evolution of vgsc mutations at domains IS6, IIS6 and IIIS6 in pyrethroid resistant* Aedes aegypti* from Mexico. Sci Rep. 2018;8:1–9, licensed under CC BY 4.0.

This correction ensures compliance with the terms of the CC BY 4.0 license and upholds the standards of transparency and proper attribution in scholarly publishing. Importantly, this correction does not affect the results, interpretations, or conclusions of the study reported in the article.
